# P46 Development of 3D in-vitro tuberculosis spheroid granuloma model using co-culture PBMCs and BEAS-2B with a nanoscaffold insert

**DOI:** 10.1093/jacamr/dlag102.052

**Published:** 2026-06-26

**Authors:** Amalia Miranda, Rocio Teresa Martinez-Nunez, Driton Vllasaliu, Jonathan Moffat, Bahijja Tolulope Raimi-Abraham

**Affiliations:** School of Cancer and Pharmaceutical Sciences, Institute of Pharmaceutical Science, King’s College London, London, UK; School of Immunology & Microbial Sciences, Department of Infectious Diseases, King’s College London, London, UK; School of Cancer and Pharmaceutical Sciences, Institute of Pharmaceutical Science, King’s College London, London, UK; Oxford Instruments Asylum Research, Halifax Road, HP12 3SE, High Wycombe, UK; School of Cancer and Pharmaceutical Sciences, Institute of Pharmaceutical Science, King’s College London, London, UK

## Abstract

**Background:**

Use of *in vitro* models in TB disease has contributed significantly to the understanding of the pathology and to drug discovery. The TB granuloma is a pathological hallmark of TB, yet replicating its structure consisting of immune and structural cells remains challenging. Peripheral blood mononuclear cells (PBMCs), comprising a diverse array of immune cells, are commonly used to study host-pathogen interactions and to construct human TB granuloma models. The BEAS-2B human bronchial epithelial cell line has been used to develop both scaffold-free and multicellular three-dimensional (3D) spheroids. The integration of natural and synthetic biomaterials in nanoscaffold fabrication has enhanced extracellular matrix (ECM) biomimicry, supporting the advancement of host-directed therapy (HDT) for infectious diseases in complex 3D models.

**Objectives:**

To develop a 3D *in vitro* TB granuloma model by co-culturing PBMCs and BEAS-2B cells with nanoscaffold inserts.

**Methods and results:**

Electrospinning was used to fabricate the nanoscaffold inserts from natural (pectin) and synthetic polymers (polyvinyl alcohol, poloxamer 407, and polycaprolactone). The hanging drop method enabled the generation of co-culture spheroids integrating PBMCs and BEAS-2B cells. Results showed that nanoscaffold inserts have a diameter below 200 nm and the Fourier Transform Infrared (FTIR) spectra confirmed the presence of each component in the nanoscaffold. It exhibited high porosity (≤70%), low moisture content (<3%), and robust nanomechanical strength. Biocompatibility assays demonstrated that nanoscaffold inserts maintained BEAS-2B viability above 75%. In the co-culture system, the optimal cell ratio was 1:1 of PBMCs:BEAS-2B cells, producing spheroids with diameters below 450 μm. The spheroids remained viable for 14 days, and the addition of nanoscaffold insert increased the structural stability. Biomarker analysis using flow cytometry confirmed the presence of PBMCs and BEAS-2B in the spheroid architecture over 14 days.

**Conclusions:**

In summary, the co-culture system successfully generated stable spheroids with the desired characteristics, and incorporating nanoscaffold inserts further improved system stability. Future experiments will include incorporation of *M. smegmatis* as a surrogate for *M. tuberculosis* to simulate TB granuloma formation. This model represents a strong candidate for further development as an *in vitro* TB granuloma platform to facilitate drug screening and host-directed therapy research.

